# Supplementary material for: (-)-5-Demethoxygrandisin B a New Lignan from Virola surinamensis (Rol.) Warb. Leaves: Evaluation of the Leishmanicidal Activity by In Vitro and In Silico Approaches
Source: Pharmaceutics. 2023 Sep 7;15(9):2292. doi: 10.3390/pharmaceutics15092292 (PMC10535778; doi:10.3390/pharmaceutics15092292)

Figure S1. Mass spectrum of the precursor ion (new (-)-5-demethoxygrandisin B) and its isotopes.

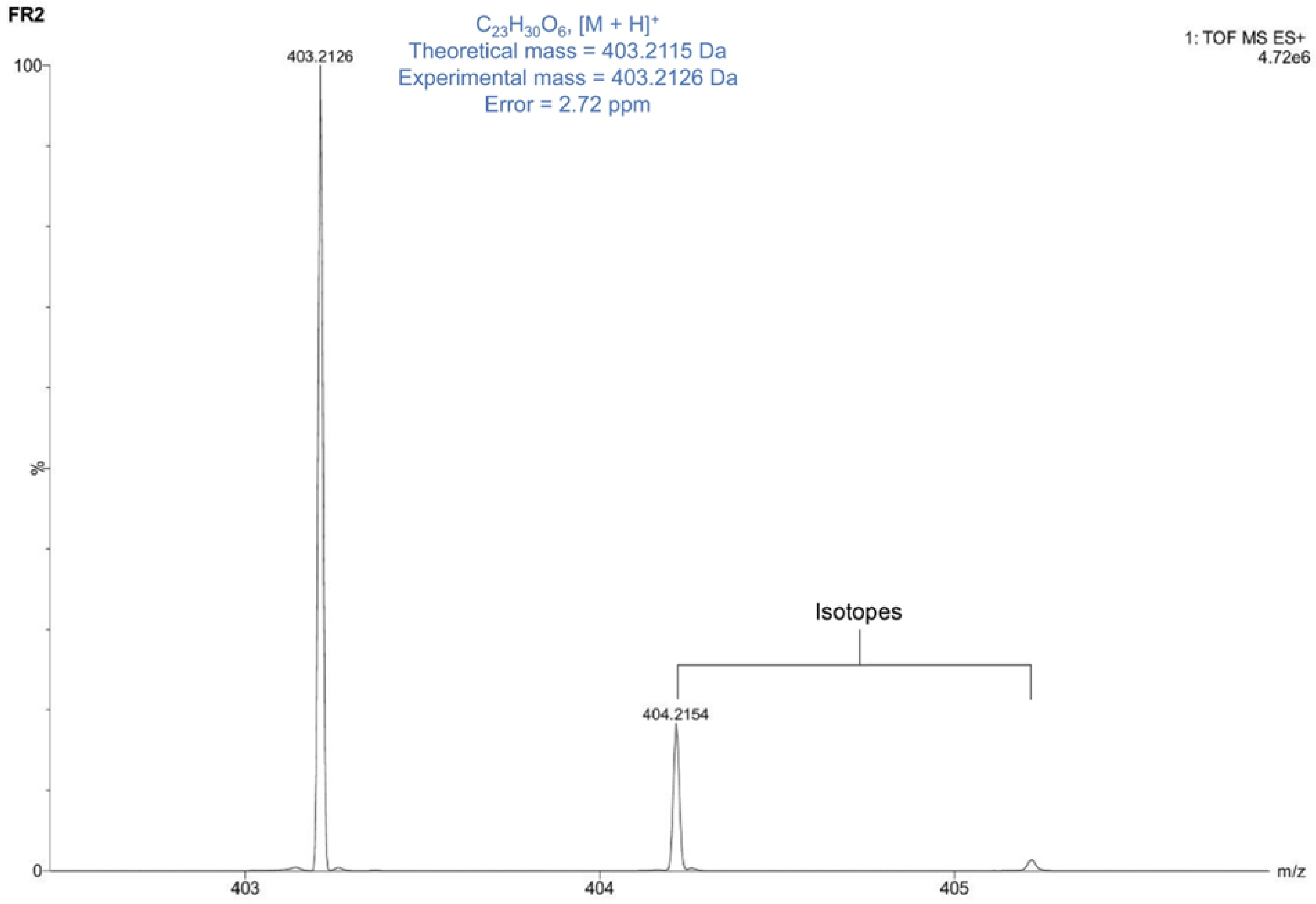

Figure S2. ^{1}H NMR for the new (-)-5-demethoxygrandisin B in $CDCl_3$ (400 MHz)

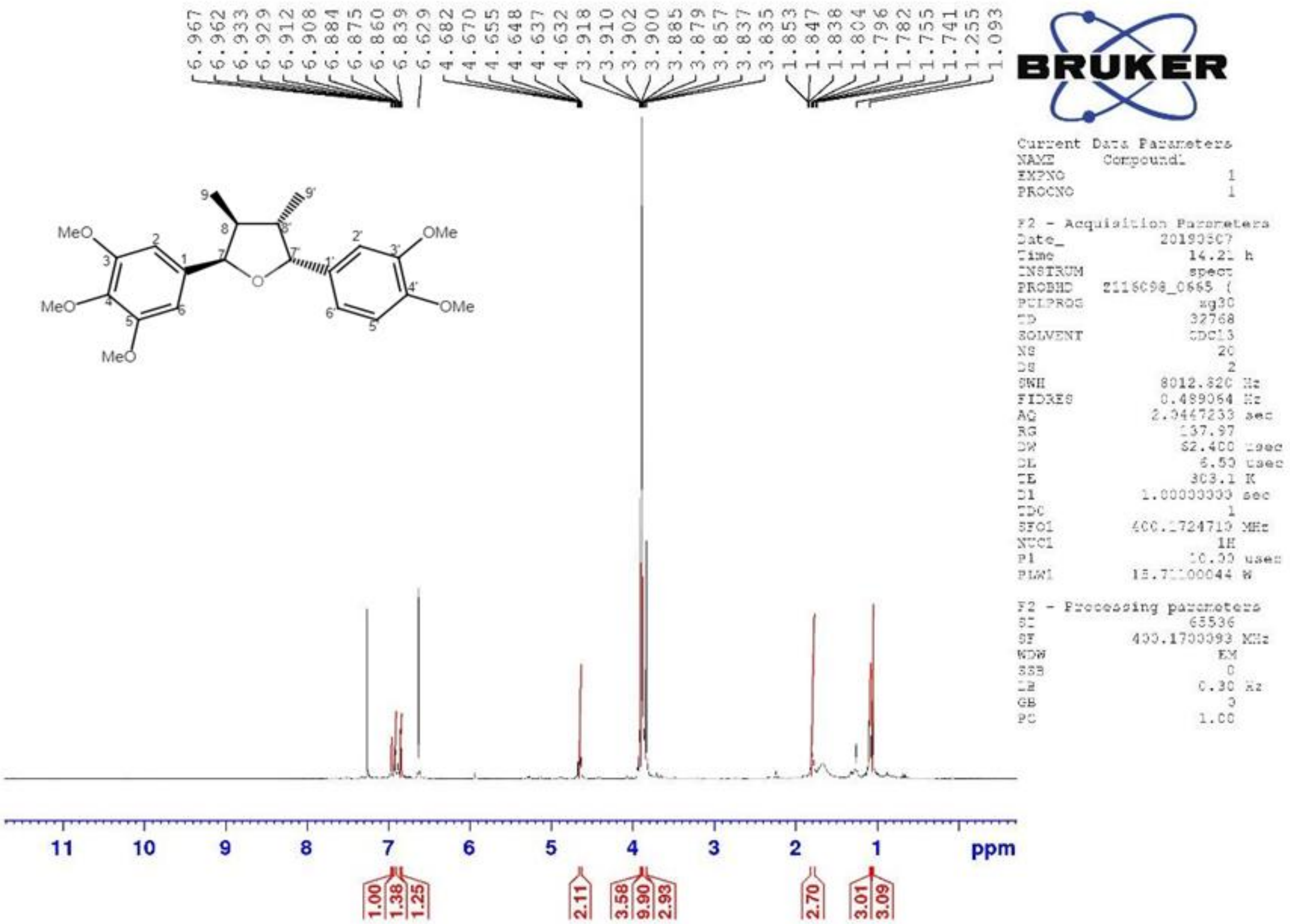

Figure S3. ^{13}C NMR for the new (-)-5-demethoxygrandisin B in $CDCl_3$ (100 MHz)

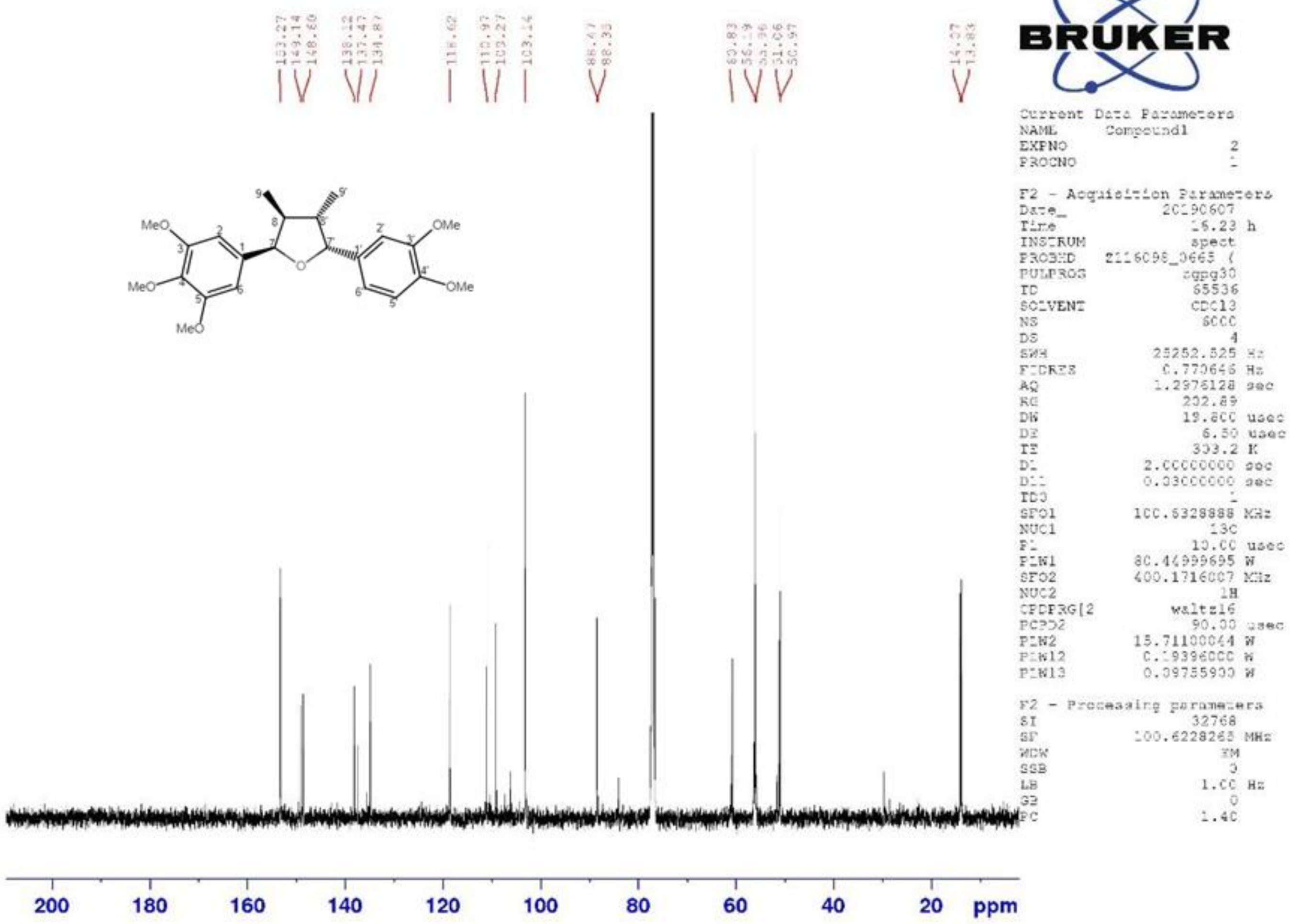

Figure S4. COSY for the new(-)-5-demethoxygrandisin B in $CDCl_3$

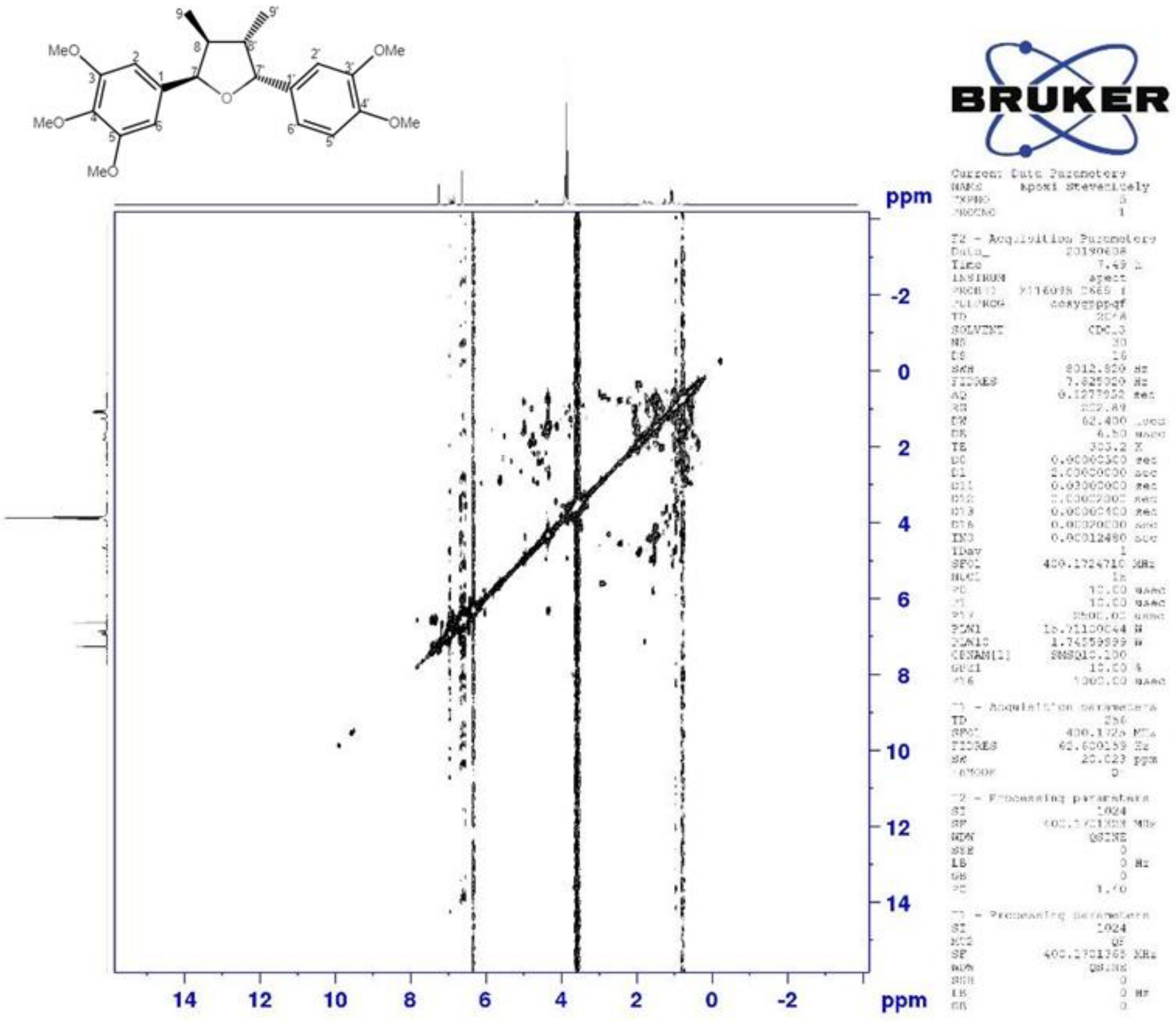

Figure S5. HSQC for the new (-)-5-demethoxygrandisin B in $CDCl_3$

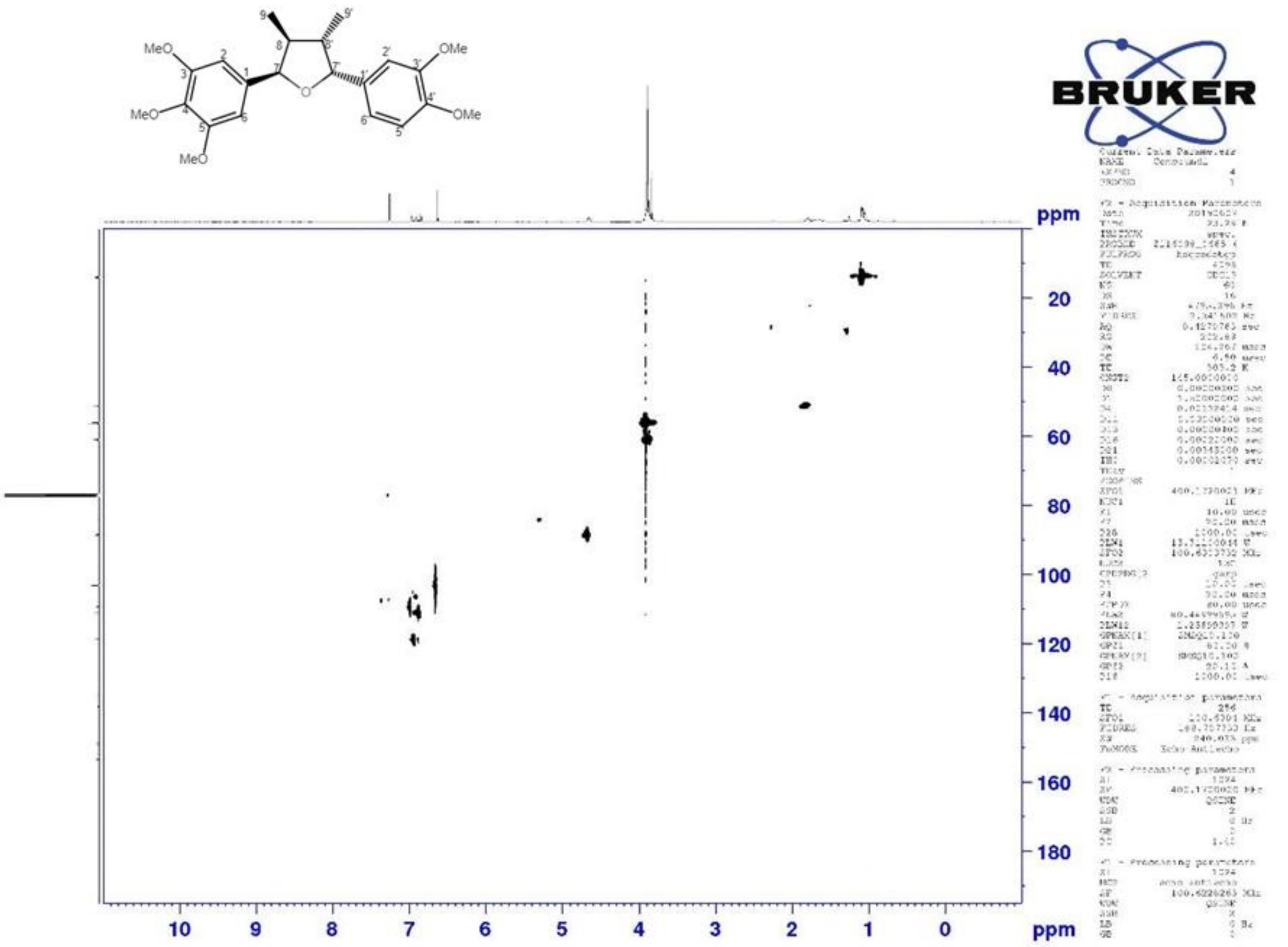

Figure S6. HMBC for the new (-)-5-demethoxygrandisin B in $CDCl_3$

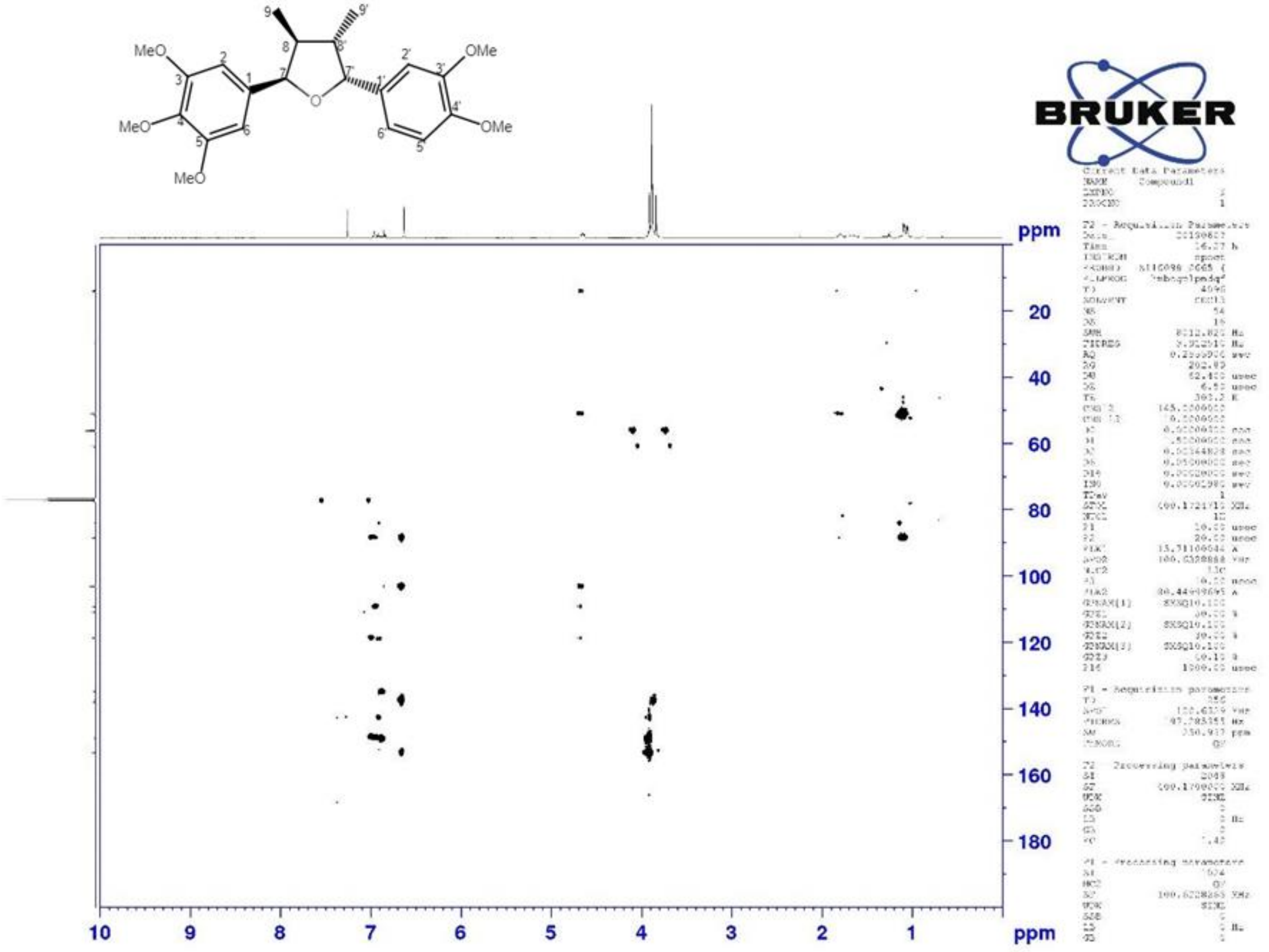

Supplement: Supplementary file 1 [file pharmaceutics-15-02292-s001.zip › pharmaceutics-2544704-supplementary.pdf]
